# Overexpression of parkin rescues the defective mitochondrial phenotype and the increased apoptosis of Cockayne Syndrome A cells

**DOI:** 10.18632/oncotarget.9913

**Published:** 2016-06-07

**Authors:** Barbara Pascucci, Mariarosaria D’Errico, Alessandra Romagnoli, Chiara De Nuccio, Miriam Savino, Donatella Pietraforte, Manuela Lanzafame, Angelo Salvatore Calcagnile, Paola Fortini, Sara Baccarini, Donata Orioli, Paolo Degan, Sergio Visentin, Miria Stefanini, Ciro Isidoro, Gian Maria Fimia, Eugenia Dogliotti

**Affiliations:** ^1^ Institute of Crystallography, Consiglio Nazionale delle Ricerche, Monterotondo Stazione, Rome, Italy; ^2^ Department of Environment and Primary Prevention, Istituto Superiore di Sanità, Viale Regina Elena, Rome, Italy; ^3^ Department Epidemiology and Preclinical Research, INMI L. Spallanzani IRCCS, Rome, Italy; ^4^ Department of Cell Biology and Neurosciences, Istituto Superiore di Sanità, Viale Regina Elena, Rome, Italy; ^5^ Laboratory of Molecular Pathology, Department of Health Sciences, Università del Piemonte Orientale, Novara, Italy; ^6^ Institute of Molecular Genetics, Consiglio Nazionale delle Ricerche, Pavia, Italy; ^7^ IRCCS Azienda Ospedaliera Universitaria San Martino-IST-Istituto Nazionale per la Ricerca sul Cancro, Largo Rosanna Benzi, Genova, Italy; ^8^ Department of Biological and Environmental Sciences and Technologies (DiSTeBA), Università del Salento, Lecce, Italy

**Keywords:** cockayne syndrome, mitochondrial dysfunction, ROS, mitophagy

## Abstract

The ERCC8/CSA gene encodes a WD-40 repeat protein (CSA) that is part of a E3-ubiquitin ligase/COP9 signalosome complex. When mutated, CSA causes the Cockayne Syndrome group A (CS-A), a rare recessive progeroid disorder characterized by sun sensitivity and neurodevelopmental abnormalities. CS-A cells features include ROS hyperproduction, accumulation of oxidative genome damage, mitochondrial dysfunction and increased apoptosis that may contribute to the neurodegenerative process. In this study, we show that CSA localizes to mitochondria and specifically interacts with the mitochondrial fission protein dynamin-related protein (DRP1) that is hyperactivated when CSA is defective. Increased fission is not counterbalanced by increased mitophagy in CS-A cells thus leading to accumulation of fragmented mitochondria. However, when mitochondria are challenged with the mitochondrial toxin carbonyl cyanide m-chloro phenyl hydrazine, CS-A fibroblasts undergo mitophagy as efficiently as normal fibroblasts, suggesting that this process remains targetable to get rid of damaged mitochondria. Indeed, when basal mitophagy was potentiated by overexpressing Parkin in CSA deficient cells, a significant rescue of the dysfunctional mitochondrial phenotype was observed. Importantly, Parkin overexpression not only reactivates basal mitophagy, but plays also an anti-apoptotic role by significantly reducing the translocation of Bax at mitochondria in CS-A cells. These findings provide new mechanistic insights into the role of CSA in mitochondrial maintenance and might open new perspectives for therapeutic approaches.

## INTRODUCTION

Cockayne Syndrome (CS) is a rare hereditary disorder with growth failure, neurological alterations and precocious aging features. Most cases of CS are caused by defects in two genes, namely *CSA* and *CSB*. CS genes are known to be involved in the repair of UV damage from the transcribed strand of active genes (transcription coupled nucleotide excision repair, TC-NER).

CSB belongs to the SWI2/SNF2 family of ATPases and, as other proteins of this family, possesses an ATP-dependent chromatin remodeling function [reviewed in 1]. The *ERCC8/CSA* gene encodes a WD-40 protein containing seven predicted repeats that act as a site for protein-protein interaction with various partners, including cullin 4A containing E3 ubiquitin ligase [2]. CSA and CSB are present in distinct protein complexes [3].

Apart of the role in TC-NER, CSA and CSB are involved in a variety of cellular pathways. CSB has been shown to interact and stimulate transcriptional protein complexes of all three classes of nuclear RNA polymerases, to regulate the re-initiation of transcription after DNA damage even in undamaged housekeeping genes, and to modulate chromatin structure (thus affecting the transcription of specific sets of genes [4]). CSA, as a subunit of an E3 ubiquitin ligase complex, interacts with CSB, driving its degradation, a step that is required for post-TC-NER recovery of transcription [5]. Moreover, CSA has been shown to interact with p44, a subunit of the RNA polymerase II basal transcription factor TFIIH [6], and to regulate the recruitment of XAB2 and HMGN1 to chromatin with stalled RNA pol II [7].

There is clear evidence that CS proteins are involved in the response to oxidative stress, and this function has been implicated in the developmental and neurological abnormalities typical of CS patients [8]. CS cells present increased levels of intracellular reactive oxygen species (ROS), an intense glycolytic metabolism, and mitochondria abnormalities [9, 10, 11]. The role of CS proteins in the response to oxidative stress is complex and multifaceted. Both nuclear CSA and CSB contribute to the repair of DNA damage caused by ROS [reviewed in 12]. Moreover, they localize at mitochondria where they interact with base excision repair BER enzymes in nucleoids [13, 14] and with proteins involved in mitochondrial transcription [15]. Lastly, CSB has been involved in the induction of mitochondrial autophagy after stress [11, 16] and in the depletion of the mitochondrial DNA polymerase γ due to deregulation of mitochondrial serine proteases [17].

It is of note that most of the information available about the mitochondrial dysfunction concerns CS-B cells. Since CSA and CSB play different roles, albeit interconnected in TC-NER of UV damage, and since CS-A and CS-B patients present similar clinical features, it is important to improve our knowledge about the role of CSA in the maintenance of mitochondrial function. Mitochondrial dysfunction and alteration in the autophagic pathways have been reported in neurodegenerative diseases [18].

Here we focused our attention on CSA. We show that CS-A cells present mitochondrial fragmentation and excessive fission. We provide evidence that the PINK1-Parkin mediated mitophagy is correctly executed in these cells, though it is insufficient to guarantee the mitochondria quality control.

By overexpressing Parkin, CS-A cells successfully recovered from mitochondrial dysfunction and were protected from apoptosis, thus suggesting Parkin as a potential therapeutic tool.

## RESULTS

### CSA deficiency is associated with increased mitochondrial fragmentation, yet with steady-state level of mitochondrial DNA oxidation

Besides the well-characterized defect in DNA repair and transcription, the functional inactivation of CSA is associated with mitochondrial dysfunction [10, 11] and hypersensitivity to oxidizing agents that target mitochondria, such as menadione (data not shown; [19]).

**Figure 1:**
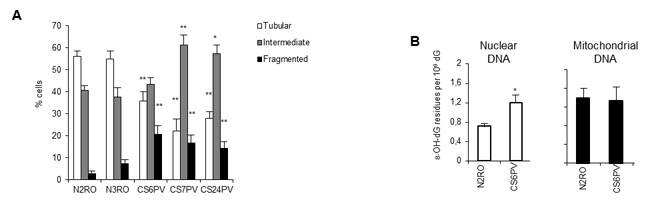
CS-A primary fibroblasts are characterized by altered mitochondrial morphology and present increased levels of oxidative damage in nuclear but not in mitochondrial DNA **A.** Distribution of cells according to mitochondrial morphology in primary fibroblasts strains from two normal (N2RO and N3RO) and three CS-A (CS6PV, CS7PV and CS24PV) donors. The reported values represent the mean ±SEM of four independent experiments; *p < 0.01, **p < 0.001 calculated within each morphological category (tubular, intermediate, fragmented) for normal *versus* CS-A cells. **B.** Basal levels of 8-OH-Gua measured as its nucleoside 8-hydroxy-2’-deoxyguanosine (8-OH-dG) by using HPLC-ED in nuclear and mitochondrial DNA of normal (N2RO) and CS-A (CS6PV) primary fibroblasts. Bars represent SD; *p < 0.05 normal *versus* CS-A cells.

In response to cellular and environmental stresses, mitochondria undergo morphological changes that are related with their function [20]. When the morphology of mitochondria was inspected with the fluorescent dye tetramethylrhodamine ethyl ester (TMRE), accumulation of damaged mitochondria was observed in primary fibroblasts of CS-A patients. As shown in Figure 1A (and Figure S1) the fraction of cells containing mitochondria with an elongated shape (tubular) is significantly higher (*p* < 0.001) in normal (N2RO, N3RO) than in CS-A (CS6PV, CS7PV, CS24PV) fibroblasts that, conversely, are enriched in fragmented mitochondria (*p* < 0.001). Since CSA, as well as CSB, is involved in the repair of nuclear oxidative DNA damage [21, 22], we first tested whether the increased abundance of fragmented mitochondria was associated with accumulation of endogenous DNA damage at mitochondria. To this aim, the steady-state level of 8-hydroxy-2’-deoxyguanosine (8-OH-Gua), a marker of cellular oxidative stress, was measured in nuclear and mitochondrial DNA of primary fibroblasts from normal and CS-A donors by HPLC-ED. An increased level of 8-OH-Gua was confirmed in nuclear DNA of CS-A (CS6PV) fibroblasts as compared to normal (N2RO) fibroblasts [21] whereas, in the case of mitochondrial DNA, the oxidation levels were indistinguishable between the two donor groups (Figure 1B).

Overall, these data indicate that the role of CSA in the protection from oxidative stress at mitochondria unlikely involves DNA repair.

### CSA interacts with DRP1 and, when defective, leads to its hyperactivation

Since the analysis in primary fibroblasts may be hampered by various factors including differences in the genetic background ([17, 23]*;* our unpublished observations), to further investigate the role of CSA in mitochondrial dynamics, we took advantage of isogenic cell lines. In particular, the study was conducted in the SV40-transformed CS-A cell line CS3BE and in its derivative CS3BE-wtCSA that stably expresses the wild-type CSA protein tagged at the C-terminus with the Flag and HA epitopes (Lanzafame et al., manuscript in preparation). The analysis of total (endogenous plus recombinant) *CSA* transcripts shows that the mRNA levels in CS3BE-wtCSA cells are two folds higher than those observed in CS3BE and in the normal MRC5 cell line, which only contains the endogenous *CSA* gene (Figure S2A). Therefore, the CS3BE-wtCSA cells constitutively express the recombinant and endogenous *CSA* at comparable levels. The presence of CSA in mitochondrial extracts [13] was demonstrated in the CS3BE-wtCSA cell line by using the HA epitope. As shown in Figure S2B, in mitochondrial as well as in the nuclear and cytosolic fractions of CS3BE-wtCSA cells we observed a band of the predicted size. No CSA specific bands were detected in the parental CS3BE cell extracts, as expected.

**Figure 2:**
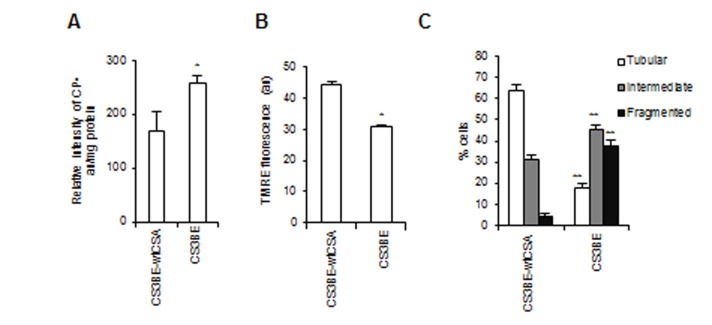
The recombinant wtCSA protein recovers the bioenergetics defect of CS3BE cells **A.** ROS levels assessed by ESR technique by measuring the intensity of the formed nitroxide 3-carboxyproxyl radical (CP^•^), and expressed in arbitrary units (au) per mg protein in the isogenic cell lines CS3BE and CS3BE-wtCSA. Experiments were performed in triplicate; *p < 0.05. **B.** Mitochondrial membrane potential expressed as arbitrary units (au) of fluorescence intensity of TMRE-loaded mitochondria in CS3BE and CS3BE-wtCSA cells. The reported values represent the mean±SEM of four independent experiments, *p < 0.001. **C.** Distribution of CS3BE and CS3BE-wtCSA fibroblasts according to mitochondrial morphology. The reported values represent the mean±SEM of four independent experiments; **p < 0.001 calculated within each morphological category (tubular, intermediate, fragmented) for CS3BE *versus* CS3BE-wtCSA cells.

Notably, the recombinant wtCSA protein is able to recover the bioenergetics defects tipically observed in CS-A primary fibroblasts [10], as well as in CS3BE cells, which are characterized by increased level of intracellular ROS measured by electron spin resonance (Figure 2A), and depolarization of the mitochondrial membrane potential (Figure 2B). Moreover, similarly to primary CS-A fibroblasts (Figure 1A), CS3BE cells present predominantly mitochondria with intermediate or fragmented shape, whereas the majority of CS3BE-wtCSA cells contain tubular mitochondria (Figure 2C, Figure S3A, S3B).

**Figure 3:**
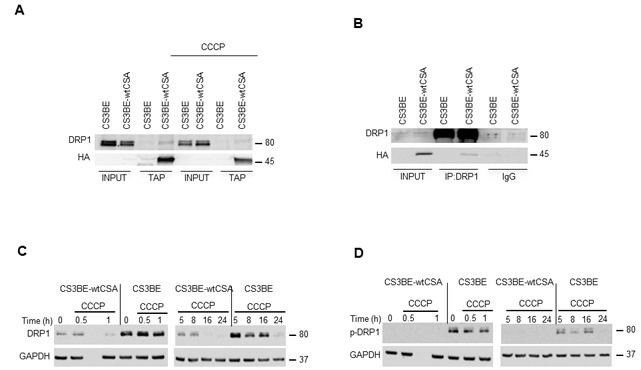
DRP1 interacts with CSA and is hyperactivated in CS-A cells **A.** Analysis of the interaction between CSA and DRP1 by TAP in the isogenic cell lines CS3BE and CS3BE-wtCSA after 20 µM CCCP treatment. **B.** Co-IP of DRP1 in the isogenic cell lines CS3BE and CS3BE-wtCSA. **C.** Analysis by western blotting of DRP1 and **D.** of its phosphorylated form at ser616 (p-DRP1) in the isogenic cell lines CS3BE and CS3BE-wtCSA after 20 µM CCCP treatment for different periods of time. Two independent experiments were performed with similar results. DRP1: 80kDa; HA-CSA: 45kDa; GAPDH: 37kDa.

Since CSA localizes at mitochondria and increased mitochondrial fragmentation is the hallmark of CS-A cells, we took advantage of the dual epitope tag of the recombinant CSA protein to check for potential interaction between CSA and the fission factor dynamin-like protein DRP1. Tandem Affinity Purification (TAP) analysis in CS3BE-wtCSA cell extracts revealed that DRP1 interacts with wtCSA both in basal condition and after exposure to the mitochondrial uncoupler carbonyl cyanide m-chloro phenyl hydrazone, CCCP (Figure 3A). Accordingly, the CSA-DRP1 interaction is observed also following DRP1 immunoprecipitation (Figure 3B).

The identification of DRP1 as a new CSA interacting protein and the altered mitochondria morphology of CS-A cells prompted us to investigate further the status of DRP1. Compared to CS3BE-wtCSA cells, CS3BE cells presented indeed an accumulation of DRP1, both in basal condition and after exposure to CCCP (Figure 3C). DRP1 phosphorylation at Ser 616 is one of the post-translational modifications known to cause the translocation of DRP1 from the cytoplasm to the mitochondria and to initiate fission [24]. Detectable levels of the activated DRP1 form (p-DRP1, phosphorylated at Ser 616) were present in CS3BE cells either untreated or under CCCP exposure but not in CS3BE-wtCSA cells (Figure 3D), indicating that hyperactivation of mitochondrial fission occurs when CSA is defective. The transcript levels of *DRP1* were consistently higher in CS3BE cells as compared to the isogenic CS3BE-wtCSA cell line. Deregulated RNA levels were also observed for the fusion proteins OPA1, MNF1 and MNF2, and for regulators of mitochondrial biogenesis such as PCG1α and β (Figure S4).

Taken together, these results further demonstrate that CSA is present in mitochondria and identify the fission protein DRP1 as a novel interacting partner of CSA. Moreover, we show that when CSA is defective DRP1 is hyperactivated, thus leading to excessive fission.

### CS-A cells present an efficient autophagic flux

The accumulation of fragmented mitochondria and the increased level of DRP1 phosphorylation might indicate that, in the absence of a functional CSA, the efficiency of authophagy as a cleaning system to get rid of dysfunctional cellular components including mitochondria is compromised.

We thus treated the cells with the mitochondrial toxin CCCP (30 µM, 1h) and analyzed by western blotting the conversion of LC3-I into its lipidated form LC3-II, a well-known marker of autophagy. The formation and accumulation of LC3-II in the presence of lysosomal inhibitors (leupeptin+NH_4_Cl), both in CS3BE-wtCSA and CS3BE cells, indicates that the basal autophagic flux is not affected in the absence of CSA (Figure S5A). Yet, the accumulation of LC3-II increases in CS3BE cells as well as in CS3BE-wtCSA cells upon treatment with CCCP (it can be appreciated in the presence of lysosomal inhibitors), indicating that autophagy is functional independently of CSA. A higher autophagic flux is noted in CS3BE cells at later recovery times. Immunofluorescence analysis showing the co-localization of LC3 (tagged with red fluorescent protein) with the mitochondrial marker OxPHOS CII strongly suggests that we are possibly observing mitochondrial autophagy (i.e., mitophagy) when CS-A cells are chronically (16h) exposed to CCCP (20 µM) (Figure S5B).

All these results indicate that CS-A cells are able to activate autophagy/mitophagy when challenged with a mitochondrial toxin such as CCCP.

### PINK1 accumulates following CCCP-induced mitochondrial depolarization in CS-A cells

**Figure 4:**
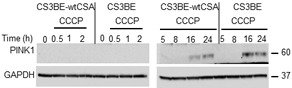
PINK is stabilized in CS-A cells under CCCP exposure Analysis of PINK1 stabilization by western blotting after 20 µM CCCP treatment for different periods of time in the isogenic cell lines CS3BE and CS3BE-wtCSA. Two independent experiments were performed with similar results. PINK1: 60kDa; GAPDH: 37kDa.

The integrity of the different steps of mitophagy was specifically addressed. Stressed mitochondria are degraded by mitophagy through a mechanism that involves PINK1 and Parkin. As shown in Figure 4, in both CS3BE-wtCSA and CS3BE cells PINK1 is stabilized (full length protein) after 16 and 24h exposure to 20 µM CCCP. After PINK1 stabilization, the next step in mitophagy is the recruitment of Parkin to damaged mitochondria. In order to monitor this step, cells overexpressing Parkin were used. We observed that, upon CCCP treatment, Parkin translocates to damaged mitochondria (marked by HSP60) (Figure S6A) that are in turn engulfed into autophagosomes as identified by LC3 (Figure S6B), providing clear evidence that the different steps of mitophagy are correctly performed even in the absence of CSA.

However, although the mitophagic process is fully functional in CS-A cells when induced by CCCP, the excessive mitochondrial fission that characterizes these cells in basal conditions is not efficiently balanced by their selective degradation *via* mitophagy.

### Overexpression of Parkin accelerates mitophagy

If Parkin mediates the selective autophagic clearance of dysfunctional mitochondria, it could be used to target damaged mitochondria to ameliorate the bioenergetics dysfunction of CS-A cells.

**Figure 5:**
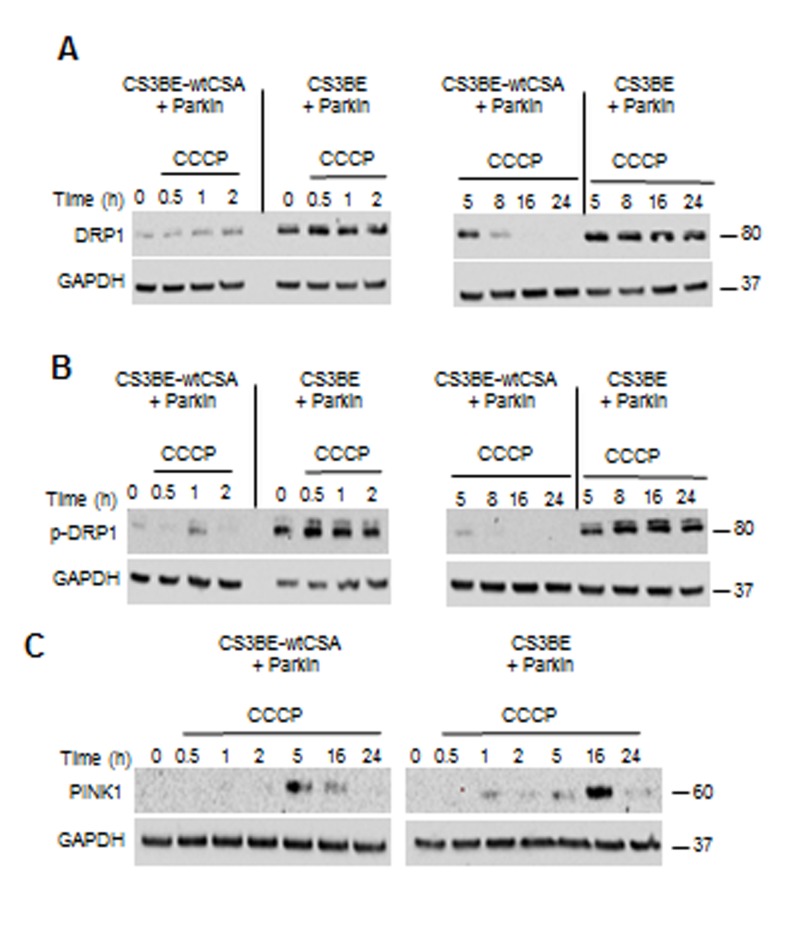
The overexpression of Parkin in CS-A cells leads to increased DRP1 phosphorylation and PINK1 stabilization **A.** Analysis by western blotting of DRP1 and **B.** of its phosphorylated form (p-DRP1) in CS3BE and CS3BE-wtCSA cells overexpressing Parkin after 20 µM CCCP treatment for different periods of time. Two independent experiments were performed with similar results. **C.** Analysis of PINK1 stabilization by western blotting after 20 µM CCCP treatment for different periods of time in CS3BE and CS3BE-wtCSA cells after Parkin overexpression. Two independent experiments were performed with similar results. DRP1: 80kDa; PINK1: 60kDa; GAPDH: 37kDa.

After CCCP treatment, an increased and persistent level of total and p-DRP1 (Figure 5A, 5B) as well as an earlier and more prolonged stabilization of PINK1 (Figure 5C) were observed in CS3BE cells overexpressing Parkin, when compared to the wtCSA-expressing counterpart. It is of note that under physiological levels of Parkin (Figure 4), PINK1 was detectable only after prolonged exposure to CCCP (16, 24h). Remarkably, under prolonged CCCP treatment the overexpression of Parkin leads to clearance of the entire mitochondrial network in both the isogenic cell lines, as indicated by the drastic decrease in the levels of the inner mitochondrial membrane markers COXIV and OXPHOS (Figure S7).

Overall these data suggest that overexpression of Parkin is able to accelerate mitophagy under CCCP exposure.

### Targeting of damaged mitochondria by Parkin overexpression rescues the defective mitochondrial phenotype of CS-A cells

**Figure 6:**
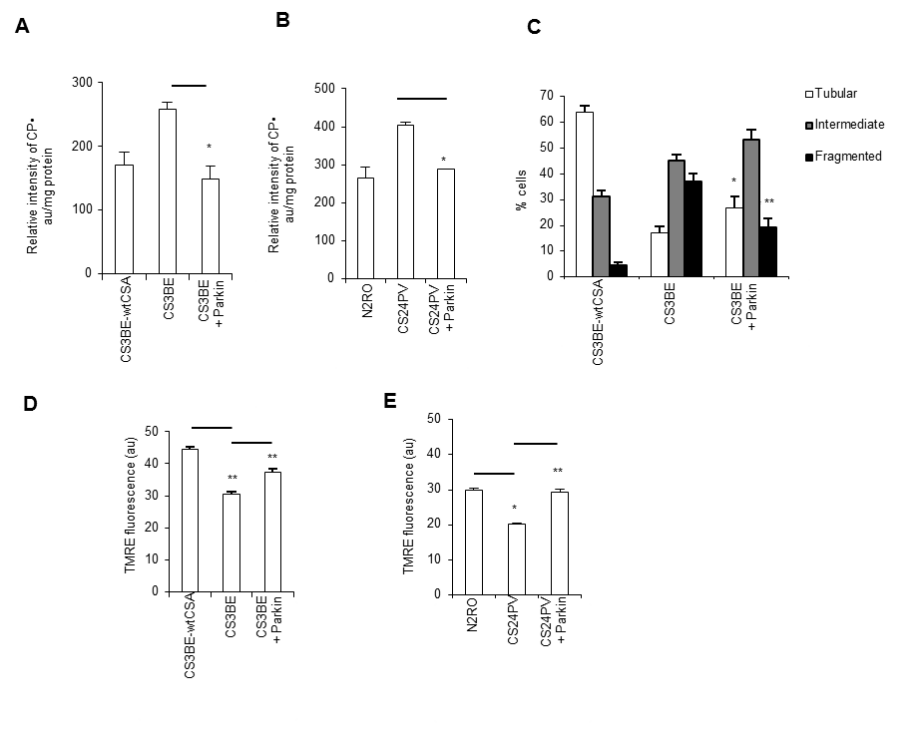
Overexpression of Parkin rescues the defective mitochondrial phenotype of CS-A cells **A.**, **B.** ROS levels assessed by ESR by measuring the intensity of the formed nitroxide 3-carboxyproxyl radical (CP^•^), and expressed in arbitraty units (au) per mg protein in **A.** CS3BE and CS3BE-wtCSA cells (*p < 0.007) and **B.** normal (N2RO) and CS-A (CS24PV) primary fibroblasts before and after Parkin overexpression (*p < 0.001). Experiments were performed in triplicate. **C.** Distribution of CS3BE-wtCSA and CS3BE cells, before and after Parkin overexpression, according to mitochondrial morphology. The reported values represent the mean ±SEM of three independent experiments; *p < 0.05, **p< 0.001 calculated within each morphological category (tubular, intermediate, fragmented) for CS3BE cells *versus* CS3BE cells after Parkin overexpression. **D.**, **E.** Mitochondrial membrane potential expressed as arbitrary units (au) of fluorescence intensity of TMRE-loaded mitochondria in **D.** CS3BE and CS3BE-wtCSA cells (**p < 0.001) and **E.** normal (N2RO) and CS-A (CS24PV) primary fibroblasts before and after Parkin overexpression (*p < 0.005, **p < 0.001). The reported values represent the mean ±SEM of four independent experiments and two independent experiments for SV40-transformed cells and primary fibroblasts, respectively.

Given the effects of overexpression of Parkin in the signaling and removal of damaged mitochondria, we hypothesized that it could be used as a tool to rescue the mitochondrial dysfunction of CS-A cells. The overexpression of Parkin was indeed able to significantly reduce ROS levels, as measured by ESR, both in the CS3BE cells and in primary fibroblasts from CS-A patients (Figure 6A, 6B), indicating that the source of ROS in CS-A cells is the dysfunctional mitochondria. This was confirmed by using the selective probe for mitochondrial superoxide MITOsox (Figure S8). Moreover, mitochondrial morphology analysis showed that Parkin overexpression in CS3BE cells is associated with a statistically significant decrease of cells containing fragmented mitochondria (Figure 6C). The overexpression of Parkin within normal cells increases the relative frequency of fragmented mitochondria by 10%, indicating that non-physiological levels of Parkin might have deleterious effects in a situation where mitochondria are not damaged (data not shown). More importantly, CS3BE cells as well as CS-A primary fibroblasts overexpressing Parkin present a recovery of mitochondrial membrane potential (Figure 6D, 6E).

**Figure 7:**
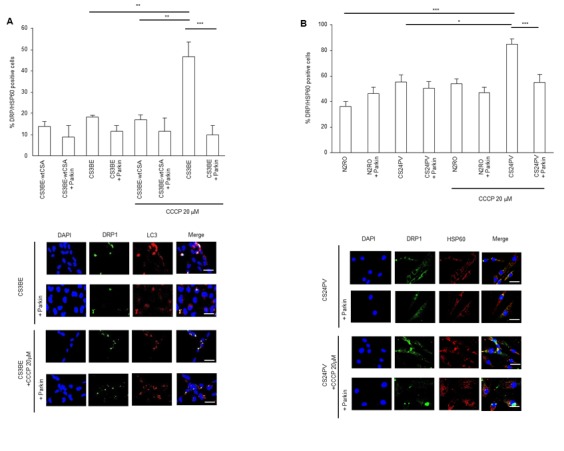
Parkin overexpression ameliorates the clearance of damaged mitochondria in CS-A cells (**A**, **B**) Top: mitochondrial DRP1 quantification (DRP1/HSP60 positive cells) in **A.** CS3BE and CS3BE-wtCSA cells (**p < 0.01, ***p < 0.001) and **B.** normal (N2RO) and CS-A (CS24PV) in primary fibroblasts (*p < 0.05, **p < 0.01, ***p < 0.001) before and after Parkin overexpression in basal conditions and after 20 µM CCCP treatment for 16h. Bottom: immunofluorescence analysis of DRP1 and LC3 **A.** and DRP1 and HSP60 **B.** localization before and after Parkin overexpression, in basal conditions and after 20 µM CCCP treatment for 16h.

Immunofluorescence analysis (Figure 7A top) showed that CS3BE cells expressing normal levels of Parkin present a trend towards increased levels of DRP1-positive mitochondria (DRP1/HSP60 positive) as compared to wtCSA expressing cells, and this increase is amplified (*p* < 0.01) upon CCCP treatment. When Parkin was overexpressed, a reduction of the DRP1-positive mitochondria was observed in CS3BE cells and this decrease was significant (*p* < 0.001) after CCCP treatment reaching levels comparable with those of wtCSA expressing cells. The same pattern was observed in CS-A primary fibroblasts (Figure 7B top and bottom). The presence of autophagosomes (identified by LC3 staining) where fragmented mitochondria are localized (DRP1-positive mitochondria) (Figure 7A bottom) testifies that in CS3BE cells, either untreated or exposed to CCCP, when Parkin is overexpressed mitophagy is taking place.

Increased activation of p53 in CS cells has been reported by several groups including ours ( [25]; reviewed in [26]), and its binding to DRP1 has been proposed as mediator of mitochondrial fission [27]. Therefore, we decided to investigate whether the silencing of p53 could also rescue the mitochondrial dysfunction of CS-A cells. To this scope, p53 was specifically silenced by shRNA in the normal (N2RO) and CS-A (CS24PV) primary fibroblasts (Figure S9A). P53 silencing did not correct the mitochondrial membrane depolarization of CS-A primary fibroblasts (CS24PV), and induced a significant decrease of the mitochondrial membrane potential in wild-type fibroblasts (Figure S9B). Thus, the homeostatic function of p53 at mitochondria seems to hamper the use of its silencing as a tool to restore healthy mitochondria.

### Parkin overexpression antagonizes the increased apoptotic rate of CS-A cells

**Figure 8:**
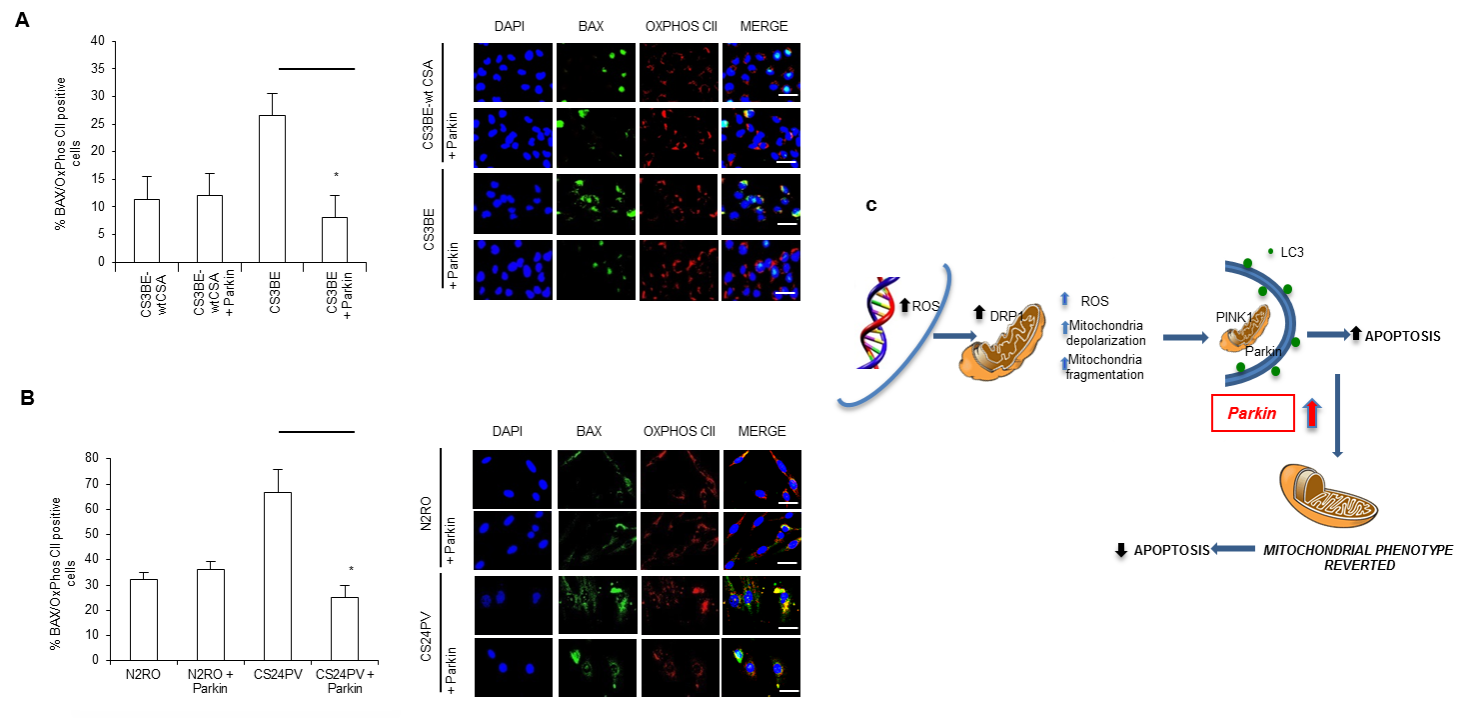
Parkin overexpression decreases apoptotic Bax at mitochondria; a model of the consequences of redox unbalance in CS-A cells **A.**, **B.** Quantification of Bax positive cells in **A.** CS3BE and CS3BE-wtCSA and **B.** normal (N2RO) and CS-A (CS24PV) in primary fibroblasts before and after Parkin overexpression; *p < 0.01. Right: immunofluorescence analysis of BAX and OXPHOS CII localization before and after Parkin overexpression. **C.** CS-A cells present increased levels of ROS/RNS. Consequently, DRP1 is hyperactivated (pDRP1) and leads to excessive fission. Parkin overexpression exerts an anti-apoptotic effect *via* elimination of the damaged mitochondria.

The phenotypic hallmark of CS cells is increased apoptosis [25, 28]. We then tested whether the overexpression of Parkin in CS-A cells, besides rescuing the defective mitochondrial phenotype, could also display an anti-apoptotic function. Bax is a regulator of apoptosis that under stress translocates from the cytosol to mitochondria. The level of Bax at mitochondria was monitored by using an antibody specific for its activated oligomeric form that occurs at the level of the mitochondria. The number of Bax-positive mitochondria was significantly higher, when CSA was defective, in both SV-40 transformed (Figure 8A) and primary fibroblasts (Figure 8B) in line with the increased susceptibility to apoptosis of CS cells. The comparison of the number of Bax-positive mitochondria in CS-A cells containing either basal or elevated levels of Parkin showed that overexpression of Parkin in CS3BE cells effectively reduced the number of Bax-positive mitochondria by 71% (Figure 8A). Similar results (reduction by 62%) were observed in primary fibroblasts (Figure 8B). The staining for vital cells unequivocally demonstrated that Parkin exerts a protective effect (Figure S10).

Taken together, all these data clearly show that Parkin is a functional anti-apoptotic tool that protects CS cells from cell death and supports cell viability *via* elimination of damaged mitochondria.

## DISCUSSION

In this study, we address the mechanism underlying mitochondrial dysfunction as observed in CS-A cells and exploit this knowledge to find a molecular tool or pathway that could reverse such defect.

### Mitochondrial DNA damage does not account for mitochondrial dysfunction

The loss of CSA as well as of CSB increases the cell sensitivity to oxidizing agents and this is in line with the accumulation of nuclear oxidative base lesions [29]. CS-A and CS-B cells are also hypersensitive to mitochondrial toxins [9, 11] and, since ROS produced at mitochondria are unlikely to reach nuclear DNA, it is expected that this hypersensitivity is due to defective repair of mitochondrial DNA. Here we show that this is not the case, since the basal level of 8-OH-Gua, a marker of oxidative DNA damage, is similar in mitochondrial DNA of CS-A as compared to normal primary fibroblasts.

Nonetheless, we observe excessive mitochondrial fragmentation in CS-A primary and transformed cells in association with a decreased mitochondrial membrane potential and excessive levels of ROS. Mitochondria with low membrane potential are more likely to be removed from the cell by autophagy. In the long term, this mechanism contributes to the maintenance of a healthy mitochondrial population and maintenance of bioenergetic capacity; however, this equilibrium is altered in CS-A cells that accumulate fragmented mitochondria.

### Mitophagy is normally executed in CS-A cells under CCCP exposure

There is some evidence that CS-B cells present defective autophagy/mitophagy [11, 16]. We have selected a consolidated system to investigate the functionality of mitophagy in CS-A cells, i.e. exposure to CCCP. It is well established [30, 31] that this mitochondrial depolarizing agent in the long-term leads to a rapid collapse of the mitochondrial membrane potential followed by a rapid accumulation of PINK1 to the outer mitochondrial membrane. The loss of mitochondrial transmembrane potential induces a translocation of Parkin from the cytosol to the mitochondria. At the outer mitochondrial membrane, PINK1 induces the ubiquitin ligase activity of Parkin by phosphorylation, thus eliciting the rapid inclusion of mitochondria in LC3-positive vacuoles and ultimately leading to their lysosomal-mediated degradation.

Notwithstanding the accumulation of fragmented mitochondria under basal conditions, we provide a clear evidence that mitophagy is correctly executed in CS-A cells upon CCCP exposure. Parkin is recruited selectively to impaired mitochondria in both normal and CS-A cells and promotes mitophagy, as shown by co-localization of LC3 with the mitochondrial marker HSP60. PINK1, normally undetectable because of its rapid cleavage and degradation [32], is no longer cleaved and becomes stabilized upon long-term exposure to CCCP, indicating increased accumulation of depolarized mitochondria in CS-A cells as compared to normal cells. This is also in line with the higher accumulation of LC3II following CCCP exposure observed in CS-A cells as compared to normal cells. It is of note that CSA cells efficiently respond to the mitochondrial uncoupling agent CCCP by activating mitophagy whereas they are unable to deal with chronic exposure to increased intracellular ROS. Therefore, as recently suggested for CS-B cells [33], the deregulation of the redox status is the Achilles’ heel of CS-A cells.

### The homeostasis of DRP1 is altered in CS-A cells

A specific feature of CS-A cells is the increased levels of endogenous total and phosphorylated (at Ser 616) DRP1. Here, we show by co-IP that CSA interacts specifically with DRP1. Activity of DRP1 is modulated by multiple post-translational modifications including phosphorylation, sumoylation, ubiquitination and S-nitrosylation [reviewed in [34]. We can exclude that the presence of CSA within the E3 ubiquitin ligase complex affects the stability of DRP1 since the ubiquitination pattern of DRP1 is unaffected by the lack of CSA (data not shown). CSA and CSB have been shown to localize at mitochondria (confirmed in this study), where they act as an anchor for BER enzymes [14]. Whether CSA regulates DRP1 homeostasis by affecting its translocation or its turnover at mitochondrial level remains to be clarified.

On the basis of the molecular features that are emerging for CS cells we can envisage alternative regulators of DRP1 homeostasis. CS proteins are involved in transcription and chromatin remodeling in response to oxidative stress [35, 1]. CSB-mediated deregulation of serine protease expression has been recently proposed to account for mitochondrial dysfunction in CS cells [17] and the translocation of DRP1 from cytosol to mitochondria is controlled by chymotrypsin-like serine proteases [36]. Moreover, the CS-dependent increased production of ROS/RNS [29, 17; this study] might be responsible *per se* of increased activation of DRP1 [37, 38]. Finally, whether the hyperactivation of p53 that characterizes CS cells plays a role by directly interacting with DRP1 [27] or with other mitophagy partners [39] deserves to be explored.

### Overexpression of Parkin “cures” the excessive apoptosis of CS-A cells

Overall, our data indicate that in CS-A cells excessive fragmentation, likely excessive fission, of mitochondria occurs and this is not balanced by mitophagy. These findings prompted us to test whether this defect could be reversed by increasing mitophagy *via* Parkin overexpression. *In vitro PARKIN* gene therapy showed a satisfactory recovery of the defective mitochondrial phenotype of CS-A cells. In the present study, we demonstrate that overexpression of Parkin in CS-A cells leads to a significant decrease of cells containing fragmented mitochondria, a reduction of the ROS levels to background and a full recovery of the mitochondrial membrane potential. We may speculate that in the absence of CSA the pathway downstream DRP1 is somehow affected and the overexpression of Parkin is able to recover an efficient mitophagic flux, as indicated by the decreased frequency of mitochondrial DRP1-positive cells under basal and CCCP treatment conditions (Figure 7A). This is in line with the promotion by Parkin of DRP1 ubiquitination and subsequent degradation at mitochondria [40]. Even more importantly, we provide evidence that Parkin displays an anti-apoptotic effect by reducing the load of activated Bax at mitochondria. Parkin-dependent regulation of Bax translocation to mitochondria, *via* ubiquitination of endogenous Bax, has been recently described [41, 42]. This central function of Parkin provides a pro-survival effect that together with selective mitophagy favors the recovery from cell injury. It is well established that CS cells are highly susceptible to spontaneous and stress-induced apoptosis [reviewed in 26]. In the brain of CS model mice (CS/XpC mice), the progressive loss of Purkinje cells (that degenerate in CS) is due to excessive apoptosis [43]. It is possible that excessive fission represents one of the early events predisposing susceptibility of CS neuronal cells to endogenous and environmental changes.

There are several examples of successful Parkin gene therapy in models of neurodegenerative diseases presenting aberrant protein aggregation and mitochondrial dysfunction. Overexpression of wild-type rat Parkin has been shown to protect against the toxicity of mutated human A30P α-synuclein in a rat lentiviral model of PD [44].

Overexpression of Parkin in APP/PS1 transgenic mice, a widely studied AD mouse model, restored activity-dependent synaptic plasticity and rescued behavioral abnormalities [45]. Interestingly, in Drosophila Parkin overexpression during aging has been shown to reduce proteotoxicity, to increase mitochondrial activity, and to extend lifespan [46]. Obviously, future research needs to focus on identifying which proteins in the pathway are specific regulators of autophagy before therapeutic targets can be selected.

In conclusion, we propose that, when CSA is defective, chronic ROS/RNS exposure leads to hyperactivation of DRP1, thus determining excessive fission/fragmentation that is “cured” by stimulating mitophagy *via* overexpression of Parkin (Figure 8C).

## MATERIALS AND METHODS

### Cell culture and treatment

Experiments were performed on primary fibroblasts obtained from biopsies from unaffected skin areas of CS-A patients (CS6PV and CS24PV) and two age-matched healthy donors (N2RO and N3RO). Cell strains were *in vitro* established and cultured as previously described [25].

A novel isogenic cell line that expresses the wild-type (wt) CSA protein tagged with the Flag and HA epitopes (CS3BE-wtCSA) was used (Lanzafame et al., manuscript in preparation). The defective counterpart is CS3BE.

The autophagy levels were assessed upon treatment by the mitochondrial uncoupler carbonyl cyanide m-chlorophenylhydrazone (CCCP, 20 µM) in DMEM with 10% serum for different time according to the experimental procedure. Lysosomal inhibitors (20 mM ammonium chloride plus 0,1 µM leupeptin) were added 2h prior to collect the cells. All reagents were purchased from Sigma (Sigma-Aldrich, Saint Louis, US).

### Measurement of 8-OH-dG by high performance liquid chromatography- electrochemical detection (HPLC-ED)

The level of 8-OH-Gua lesions was determined by HPLC-ED according to established procedures.^21^ After enzymatic digestion of DNA with nuclease P1 (Boehringer Mannheim, Germany) and alkaline phosphatase (Boehringer Mannheim, Germany), aliquots of the DNA hydrolysate were analyzed.

### Intracellular ROS levels

The intracellular ROS levels were measured by ESR technique. The spin probe 1-Hydroxy-3-Carboxy-Pyrrolidine (CPH; 0.5 mM) was added to 20x10^6^ cells/ml in phosphate buffer, pH 7.4. Samples were drawn up into a gas-permeable Teflon tube, and inserted into a quartz tube. ESR spectra were measured in air at 37°C on a Bruker ECS 106 spectrometer (Bruker, Rheinstetten, Germany) equipped with a variable-temperature unit (ER4111VT) and a ESR cavity (4108 TMH). Spectra were acquired exactly 20 min after the addition of the spin probe at 37°C. The oxidation of CPH was monitored by the formation of the characteristic 3-line spectrum with hyperfine coupling constant of 1.63 ± 0.04 mT attributable to the corresponding nitroxide radical 3-carboxyproxyl (CP^●^). Spectrometer conditions common to all spectra were: modulation frequency, 100 kHz; microwave frequency, 9.4 GHz; microwave power, 20 mW; gain 1x10^4^; modulation amplitude, 0.1 mT; conversion time, 20.5 ms; time constant, 82 ms; sweep time, 21 s; number of scans, 1.

### Immunofluorescence analysis

For confocal microscopy, cells were grown on coverslip and fixed with 4% paraformaldehyde in PBS, washed three times and permeabilized with 0.2% Triton X-100 in PBS. Proteins of interest were detected using primary antibodies diluted in PBS 1% BSA: HSP60, 1:100 (Cell Signaling-Merck Millipore, Darmstadt, Germany), OxPHOS CII, 1:1000 (Thermo Fischer Scientific, Carlsbad, US), TOMM20, 1:100 (Santa Cruz Biotechnology, California, US). HA-PARKIN was visualized upon retroviral infection by using the monoclonal antibody 12CA5. Anti-DRP1 (BD Transduction Laboratories, Erembodegem, Belgium) was used at 1:100, anti-LC3 (Sigma-Aldrich, Saint Louis, US) was used at 1:1000. Apoptosis was morphologically assessed on the basis of Bax staining using an antibody against the conformational active form (1:50, Merck Millipore, Darmstadt, Germany). Primary antibodies were incubated for 1 h at room temperature and visualised by means of Alexafluor 488 (Thermo Fischer Scientific, Carlsbad, US), Alexafluor 647 (Thermo Fischer Scientific, Carlsbad, US) e Cy3-conjugated (Jackson ImmunoResearch, West Grove, US) secondary antibodies (1:500). Nuclei were stained with DAPI (BD Transduction Laboratories, Erembodegem, Belgium ).

Coverslips were mounted using the SlowFade solution (Thermo Fischer Scientific, Carlsbad, US) and examined under a confocal microscope (TCS SP2; Leica, Wetzlar, Germany) or with a Leica DMI600 fluorescence microscope (Leica, Wetzlar, Germany).

For each experimental condition three cover-slips were prepared. At least five fields in each cover-slip were imaged and examined by two independent investigators. Representative images of selected fields are shown.

### Mitochondria morphology analysis

To measure mitochondrial morphology the potentiometric dye tetramethylrhodamine ethyl ester perchlorate (TMRE) was used at a final concentration of 30 nM (from 1 mM stock solution in DMSO). Cells were kept for 30 min in the presence of TMRE before recording, in order to reach saturation of the dye, and maintained throughout the entire experiment to avoid the decay of the signal. The presence of ROS at mitochondria was assayed with MITOSox-redTM (Thermo Fischer Scientific, Carlsbad, US), which essentially detect superoxide anion produced by mitochondria [47]. For MITOSox loading , cells were kept for 60 min in a saline solution containing 5 µM of the dye.

The saline solution used for loading and recording had the following composition: NaCl 140 mM, KCl5 mM, CaCl2 2.5 mM, MgCl2 1 mM, D-glucose 10 mM, HEPES/NaOH 10 mM (RT, pH 7.4, 290 mosmol l-1).

An oil immersion objective (40X, 1.35 NA, Olympus, Tokyo, Japan) mounted on an inverted microscope (Axiovert 135, Zeiss, Oberkochen, Germany) was utilized for fluorescence video imaging. For both dyes, TMRE and MITOSox, an excitation wavelength of 535 nm was applied by means of a monochromator (Till Photonics, Polychrome II; Germany) and the emission light at 590 nM was collected by a CCD, cooled digital camera (PCO, Sensicam; Germany) and recorded on the hard disk of a PC computer. The Imaging Workbench 6.0 software package (Indec BioSystems, Santa Clara, US) was used for recording and off-line analysis of the data. The software allowed the measurement of the emission values also along line profiles crossing mitochondria. A minimum of two peaks of amplitude was used to calculate the average amplitude of a given mitochondrion. Since the organelles were densely packed around a mitochondria-free area corresponding to the nucleus and single mitochondria were detectable only in the periphery of a cell, only mitochondria in this latter area were chosen for analysis. TMRE-loaded cultures were also used to evaluate mitochondrial morphology and to classify the cells accordingly.

### Quantitative real time RT-PCR

Total RNA was purified using the “RNeasy Mini Kit” (Quiagen, Hilden, Germany), according to manufacturing instructions. 1 µg of RNA was reverse-transcribed using the iScript Reverse Transcriptase kit (Biorad, CA, US). The cDNAs were diluted 1:10 in water and 3 µl were used as template in real time RT-PCR reactions containing 10 pmol of the specific primer pairs (CSA-F1: GACTATATCTTGGCAACAG; CSA-R1: GTGACTTTTTCCCATTATGT; GAPDH-F2: GAGTCAACGGATTTGGTCGT; GAPDH-R2: GACAAGCTTCCCGTTCTCAG). The real time reactions were performed using the LightCycler 480 Real-Time PCR System (Roche Diagnostics, Milan, Italy). Standard curves were obtained for each primer set with serial dilutions of cDNA and the relative amount of each mRNA was normalized to the relative amount of the housekeeping gene GAPDH.

In order to quantify transcript levels of mitochondrial proteins RNA was extracted by RNeasy kit (Quiagen, Hilden, Germany) and cDNA synthesis was carried out by the high capacity cDNA reverse transcription kit (Thermo Fischer Scientific, Carlsbad, US). Gene expression analysis was carried out using single tube Taqman real-time PCR assays (Thermo Fischer Scientific, Carlsbad, US).

### Protein expression analysis

Cell homogenates were prepared by ultrasonication in a buffer containing detergents and proteases and phosphatases inhibitors (Complete EDTA-free protease inhibitor cocktail and Phos-STOP phosphatase inhibitor cocktail, Roche Diagnostics, Milan, Italy). Mitochondrial fractionation was performed by using a specific kit (Active Motif, Carlsbad, US) according to the manufacturer’s instructions. Proteins were separated on NuPAGE 4-12% precast polyacrylamide gels (Thermo Fischer Scientific, Carlsbad, US) and analysed by western blotting with the following antibodies: rabbit polyclonal anti-LC3 (Merck Millipore, Darmstadt, Germany), rabbit polyclonal anti-PINK1 (Novus Biologicals, Littleton, US), mouse monoclonal anti-DRP1 (BD Transduction Laboratories, Erembodegem, Belgium), rabbit monoclonal anti-Phospho-DRP1 (Ser616) (D9A1) (Merck Millipore, Darmstadt, Germany), rabbit polyclonal anti-p53 (FL-393) (Santa Cruz Biotechnology INC, US), mouse monoclonal anti-complex II 70 KDa Fp subunit antibody (Thermo Fischer Scientific, Carlsbad, US), rabbit polyclonal anti-Cox IV antibody (Merck Millipore, Darmstadt, Germany), rat monoclonal anti-HA (3F10) (Roche Diagnostics, Milan, Italy), mouse monoclonal anti-PCNA (PC10) (Santa Cruz Biotechnology INC, US), mouse monoclonal anti-GAPDH (6C5) (Santa Cruz Biotechnology INC, US), rabbit polyclonal anti-TOMM20 (Sigma-Aldrich, Saint Louis, US). Immunocomplexes were revealed by using a peroxidase-conjugated secondary antibody (Biorad, CA, US). Quantitation of protein bands was performed by using Chemidoc (Biorad, CA, US).

### Tandem affinity purification (TAP)

Total extracts from 2x10^8^ cells were incubated o.n. at 4°C with anti-Flag^®^ M2 Affinity Gel (Sigma-Aldrich, Saint Louis, US). Beads were washed 5 times with 1X IP low buffer (Active Motif, Carlsbad, US) and the immunoprecipitated proteins were eluted with the Flag peptide (Sigma-Aldrich, Saint Louis, US) in TBS (Tris 10 mM pH 7.4, NaCl 150 mM). Eluates were further immunoprecipitated with anti-HA agarose affinity resin (Sigma-Aldrich, Saint Louis, US) o.n. at 4°C. Beads were washed 5 times with 1X IP low buffer and the immunoprecipitated proteins were eluted by incubating the beads for 10 minutes at RT with 2X NuPAGE-LDS Sample Buffer (Thermo Fischer Scientific, Carlsbad, US). Eluates were supplemented with 50 mM DTT and further investigated by immunoblotting.

### Protein immunoprecipitation

Total extracts from 2x10^7^ cells were immunoprecipitated with anti-Drp1 antibody (BD Transduction Laboratories, Erembodegem, Belgium) according to standard protocols. Briefly, extracts were pre-cleared with protein A/G agarose beads, incubated o.n. at 4°C with 5µg of antibody and subsequently with protein A/G agarose beads for 2 hours. Beads were washed 3 times with 1X IP high buffer (Active Motif, Carlsbad, US) supplemented with 1 mg/ml BSA and 3 times with 1X wash IP high buffer. Immunoprecipitated proteins were eluted for 10 minutes at 70°C with NuPAGE-LDS Sample Buffer 2X (Thermo Fischer Scientific, Carlsbad, US), supplemented with 50 mM DTT and further investigated by immunoblotting.

### Lentivirus and retrovirus generation and infection

HA-Parkin cDNA was subcloned from the pRK5-HA-Parkin (Plasmid #17613 Addgene, Cambridge, UK) to the pLPCX vector (Clontech, CA, US) for retroviral infection by EcoRI-NotI double digestion. pLPCX RFP-LC3 was previously decribed [48]. 15 µg of retroviral vectors were cotransfected with 5 µg expression plasmid for the vesicular stomatitis virus G protein into 293 gp/bsr cell line by using the calcium phosphate method. After 48h, the supernatant containing the retroviral particles was recovered and supplemented with 4μg/ml polybrene. For infection, cells were incubated with retrovirus-containing supernatant for 6h.

For stable p53 RNA interference, a lentiviral pLKO.1 plasmid targeting p53 mRNA was used (Addgene plasmid # 19119, Cambridge, UK). The lentiviral production was obtained by co-transfecting pMDLg and psPAX2 plasmids in HEK293T and supernatants were collected 48h post-transfection. For infection, cells were incubated with lentivirus-containing supernatant for 6h.

## SUPPLEMENTARY MATERIALS FIGURES


