# Supplementary material for: Overexpression of parkin rescues the defective mitochondrial phenotype and the increased apoptosis of Cockayne Syndrome A cells
Source: Oncotarget. 2016 Jun 7;8(61):102852–67. doi: 10.18632/oncotarget.9913 (PMC5732694; doi:10.18632/oncotarget.9913)
Supplement: Supplementary file 1 [file oncotarget-08-102852-s001.pdf]

Overexpression of parkin rescues the defective mitochondrial phenotype and the increased apoptosis of cockayne syndrome A cells

Supplementary Material

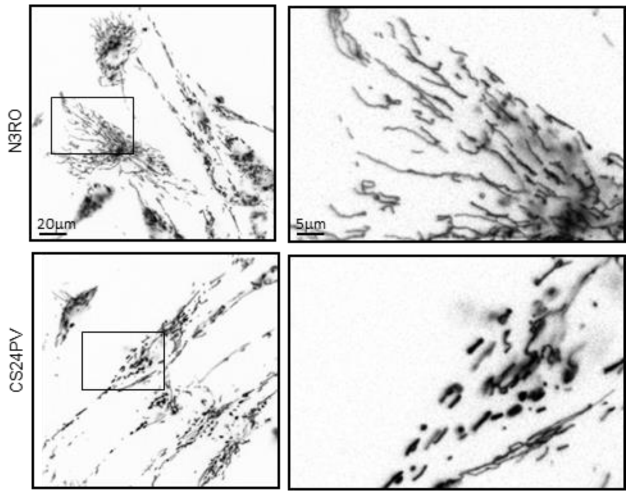

Supp. Fig. 1: **CS-A primary fibroblasts present an altered mitochondrial morphology.**

Photomicrographs showing TMRE-loaded mitochondria from normal (N3R0) and CS-A (CS24PV) primary fibroblasts (Calibration bar=20 and 5µm).

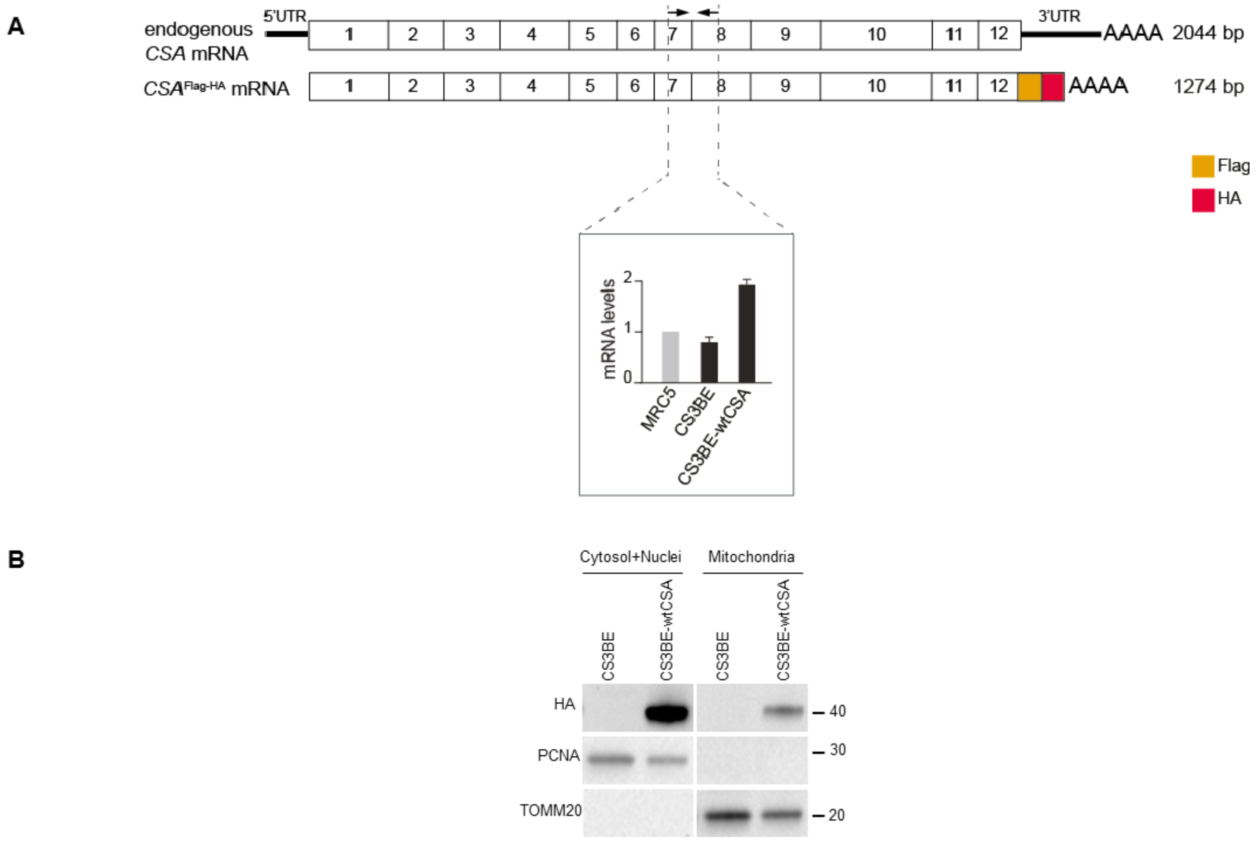

Supp. Fig. 2: **The recombinant *CSA*^{FlagHA} mRNA is expressed at levels comparable to endogenous *CSA* and the encoded protein localizes also to mitochondria**.

(A) Total (endogenous and recombinant) *CSA* expression levels in the isogenic cell lines CS3BE and CS3BE-wtCSA and in the normal MRC5 cell line evaluated by quantitative real time RT-PCR. mRNA levels were first normalized to *GAPDH* expression levels and afterwards to the levels in normal MRC5 cells. The reported values represent the mean of three independent experiments. The upper panel is a schematic representation of the endogenous *CSA* and recombinant CSA^{FlagHA} transcripts showing the coding exons (numbered rectangles) and the position of PCR primers (arrows). (B) Analysis by western blotting using an antibody to the HA epitope in mitochondrial and cytosolic extracts of CS3BE and CS3BE-wtCSA cells. Antibodies against PCNA and TOMM20 were used as marker for cytosolic/nuclear and mitochondrial fractions, respectively. HA-CSA: 45kDa; PCNA: 30kDa; TOMM20: 20kDa

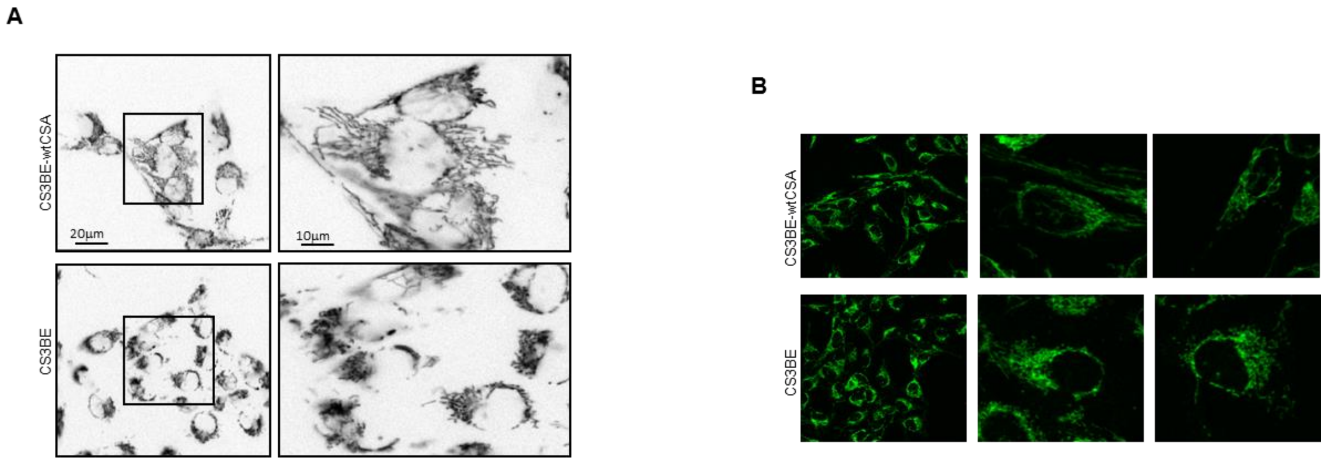

Supp. Fig. 3: **CS3BE cells present an altered mitochondrial morphology that is recovered following wtCSA expression.**

(A) Photomicrographs showing TMRE-loaded mitochondria from the isogenic cell lines CS3BE and CS3BE-wtCSA (Calibration bar=20 and 10µm). (B) Analysis of mitochondrial shape in CS3BE and CS3BE-wtCSA cell lines with confocal microscopy using an antibody against TOMM20.

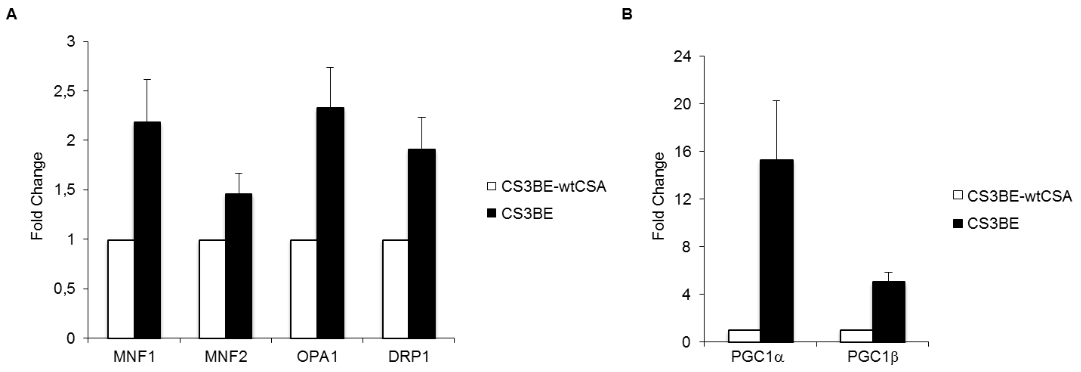

Supp. Fig. 4. **Altered expression levels of mitochondrial fusion, fission and biogenesis genes in CS3BE cells.**

Analysis by real time PCR of mRNA levels of the fusion (MFN1, MFN2, OPA), fission (DRP1) and biogenesis (PGC1α, PGC1β genes in the isogenic cell lines CS3BE and CS3BE-wtCSA.

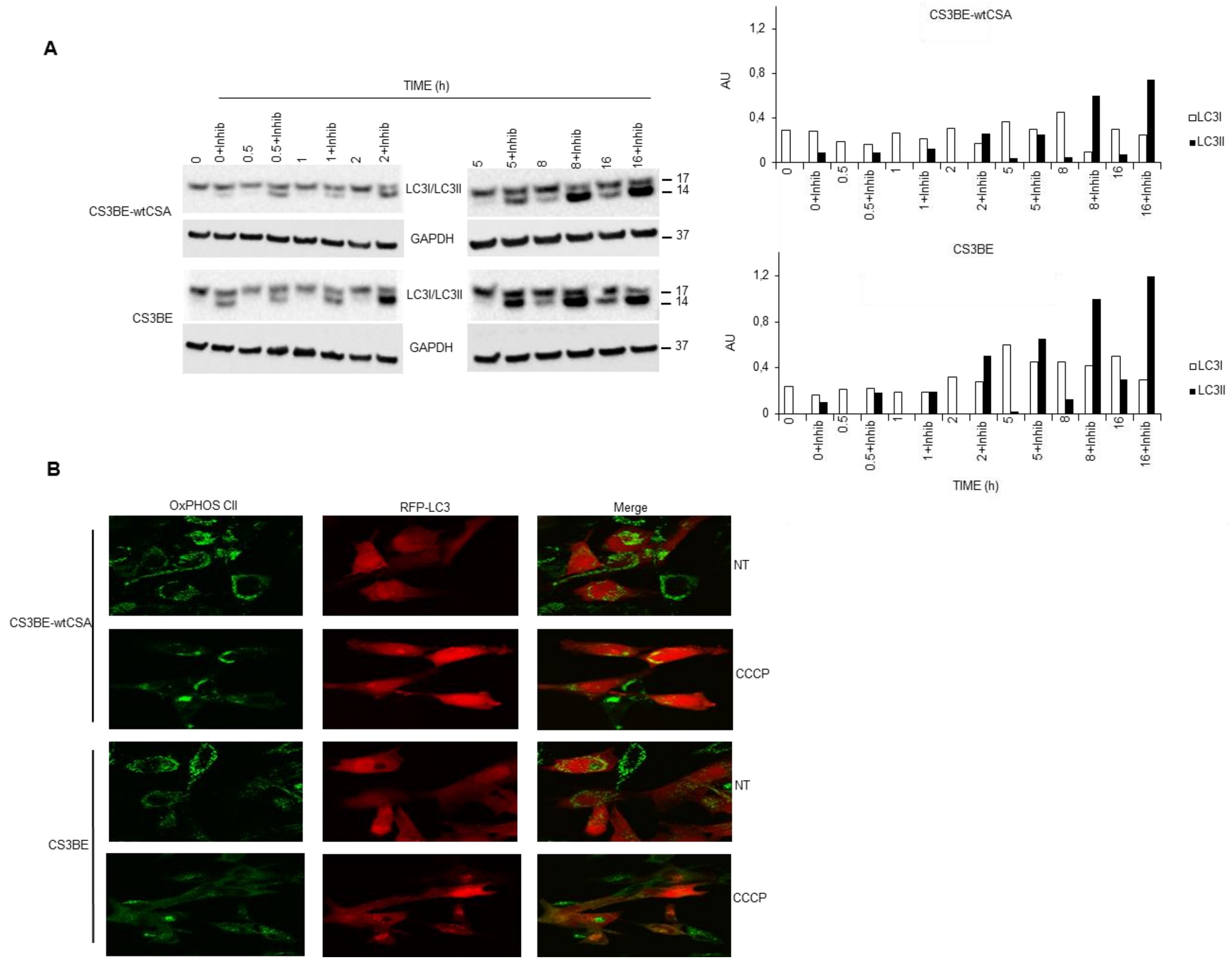

Supp. Fig. 5: **CS-A cells present an increased autophagic flux.**

(A) Left: Analysis by western blotting of LC3I and LC3II in the isogenic cell lines CS3BE and CS3BE-wtCSA after 1h exposure to 30 μM CCCP at different recovery times in the presence or absence of lysosomal inhibitors (inhib=NH_4Cl+leupeptin). The third lane of the original western blotting of CS3BE cells (left panel) has been removed since it was an empty lane. Right: Densitometric analysis. Two independent experiments were performed with similar results. (B) Colocalization of LC3 (autophagosome marker) tagged with red fluorescent protein (RFP-LC3) with OxPHOS CII (mitochondrial marker) in untreated or 30uM CCCP treated CS3BE and CS3BE-wtCSA cells. LC3I/LC3II:17/14kDa; GAPDH: 37kDa.

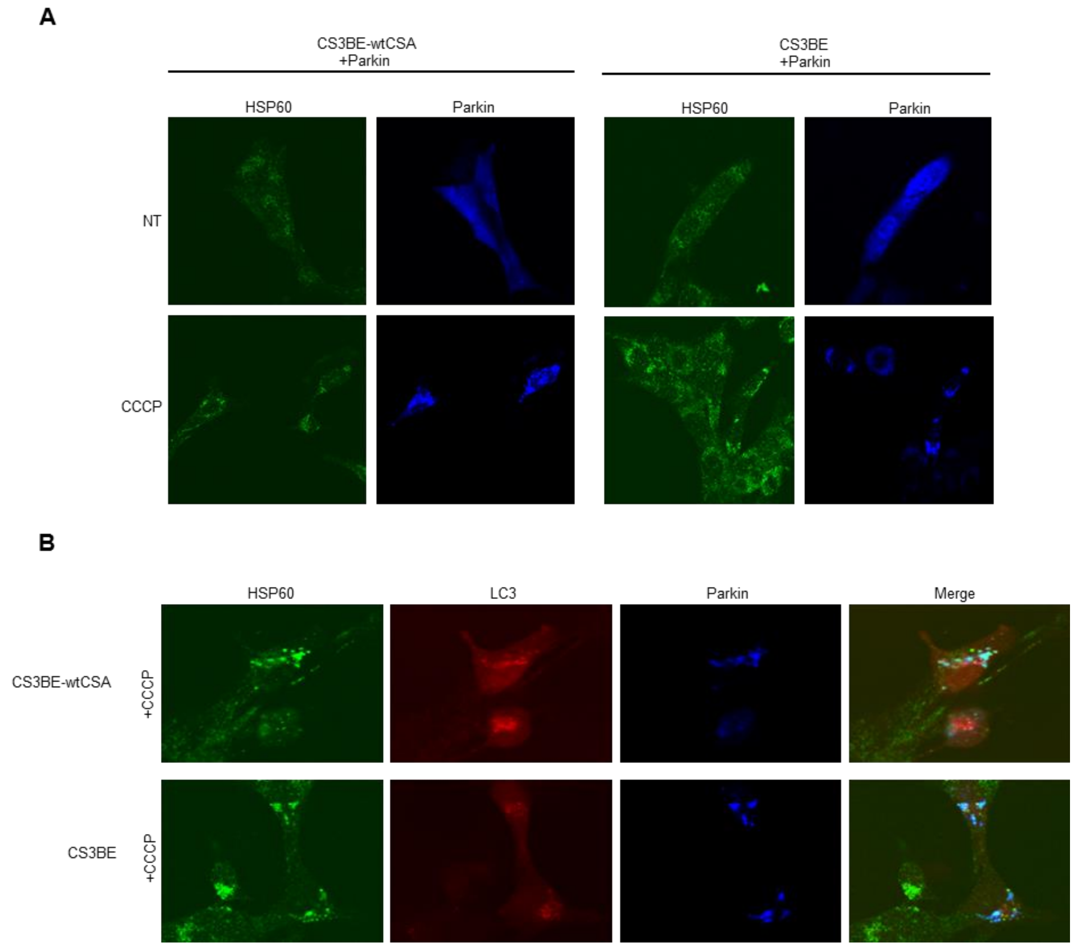

Supp. Fig. 6: **Parkin traslocates to mitochondria in CS-A and wild-type CSA expressing cells after CCCP treatment.**

(A) Analysis by immunofluorescence of Parkin translocation in untreated (NT) or CCCP-treated (20 μM CCCP for 16h) CS3BE and CS3BE-wtCSA cells overexpressing Parkin (CS3BE+Parkin, CS3BE-wtCSA+Parkin). Anti-heat shock protein 60 (HSP60) antibody was used to mark the mitochondria. (B) Immunofluorescence analysis of Parkin and localization with the autophagic marker LC3 in the isogenic cell lines CS3BE and CS3BE-wtCSA. HSP60 antibody was used to mark the mitochondria.

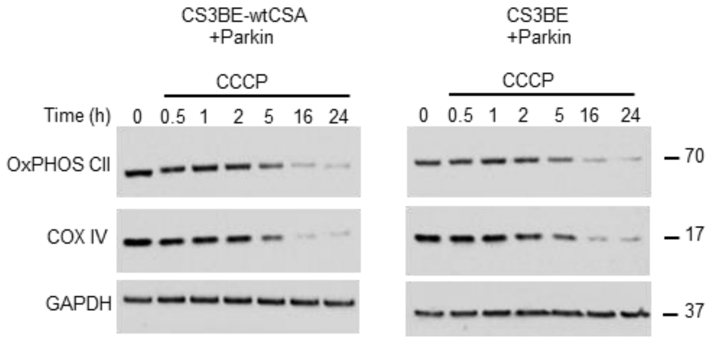

Supp. Fig. 7: **Parkin overexpression leads to the clearance of the mitochondrial network.**

Analysis by western blotting of COXIV and OXPHOS after 20 μM CCCP treatment for different periods of time in CS3BE and CS3BE-wtCSA cells after Parkin overexpression. Two independent experiments were performed with similar results. OXPHOS:70kDa; COXIV: 17kDa; GAPDH: 37kDa.

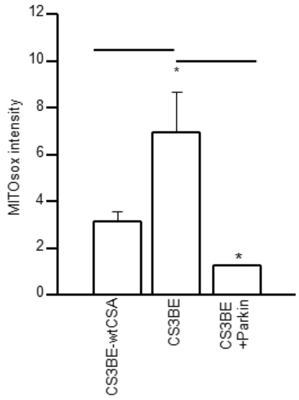

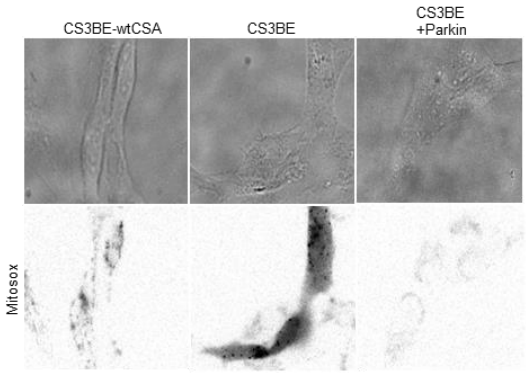

Supp. Fig. 8: **Parkin overexpression reduces mitochondrial ROS in CS-A cells.**

Mitochondrial ROS quantification (top) and, immunofluorescence analysis by MITOsox (bottom) in CS3BE-wtCSA and CS3BE cells before and after Parkin overexpression; *p <0.05.

.

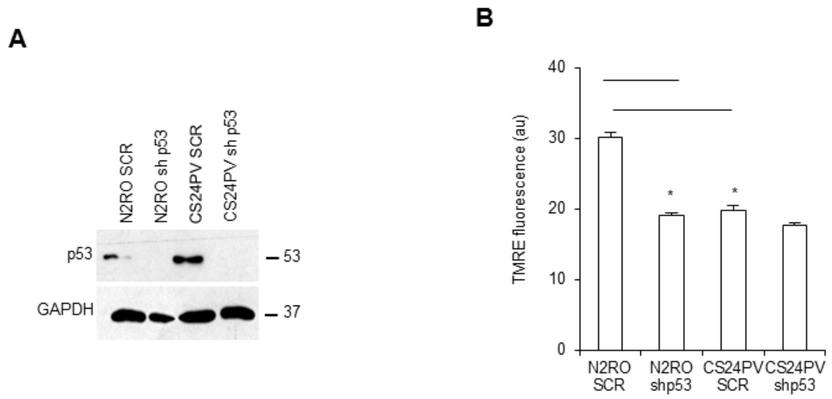

Supp. Fig. 9: **Effects of p53 silencing on mitochondria**

(A) Analysis by western blotting of p53 silencing in normal (N2RO) and CS-A (CS24PV) primary fibroblasts. (B) Mitochondrial membrane potential expressed as arbitrary units (au) of fluorescence intensity of TMRE-loaded mitochondria in normal (N2RO) and CS-A (CS24PV) primary fibroblasts following p53silencing. The reported values represent the mean±SEM of three independent experiments; *p<0.001. SCR (scrambled) shp53 (short harpin p53). P53: 53kDA; GAPDH: 37kDa.

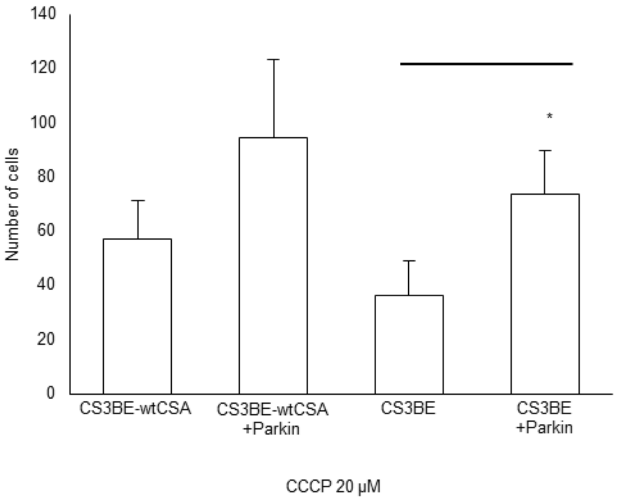

Supp. Fig. 10: **Parkin exerts a protective effect on cell survival.**

Quantification of DAPI positive cells after CCCP treatment (20 μM for 16h) in CS3BE-wtCSA and CS3BE cells before and after Parkin overexpression. *p <0.05.
